# Microbial Electrochemical Systems: Principles, Construction and Biosensing Applications

**DOI:** 10.3390/s21041279

**Published:** 2021-02-11

**Authors:** Rabeay Y.A. Hassan, Ferdinando Febbraio, Silvana Andreescu

**Affiliations:** 1 Nanoscience Program, University of Science and Technology (UST), Zewail City of Science and Technology, 6th October City, Giza 12578, Egypt; ryounes@zewailcity.edu.eg; 2 National Research Centre (NRC), Applied Organic Chemistry Department, El Bohouth st., Dokki, Giza 12622, Egypt; 3 Institute of Biochemistry and Cell Biology, National Research Council (CNR), Via P. Castellino 111, 80131 Naples, Italy; ferdinando.febbraio@cnr.it; 4 Department of Chemistry and Biomolecular Science, Clarkson University, Potsdam, NY 13699-5810, USA

**Keywords:** microbial electrochemical systems, bioelectrochemistry, biosensing devices, electrode materials, electron transfer

## Abstract

Microbial electrochemical systems are a fast emerging technology that use microorganisms to harvest the chemical energy from bioorganic materials to produce electrical power. Due to their flexibility and the wide variety of materials that can be used as a source, these devices show promise for applications in many fields including energy, environment and sensing. Microbial electrochemical systems rely on the integration of microbial cells, bioelectrochemistry, material science and electrochemical technologies to achieve effective conversion of the chemical energy stored in organic materials into electrical power. Therefore, the interaction between microorganisms and electrodes and their operation at physiological important potentials are critical for their development. This article provides an overview of the principles and applications of microbial electrochemical systems, their development status and potential for implementation in the biosensing field. It also provides a discussion of the recent developments in the selection of electrode materials to improve electron transfer using nanomaterials along with challenges for achieving practical implementation, and examples of applications in the biosensing field.

## 1. Introduction

Microbial electrochemical system (MES) is a promising fast expanding technology that integrates microbial systems, electrochemistry and materials science to develop energy, environment and sensing devices [1]. MES exploits the biocatalytic activity of living microbes to harvest electrons from the biodegradable organic substances and therefore explore the interaction between living microbial cells (electron donor) and surface of electrodes (electron acceptor) [2]. A critical requirement for the development of MESs is to achieve effective integration and facilitate electron transfer between microorganisms (e.g., having ability to accept or donate electrons to and from electrodes) and the electrode surface, which are fundamental to their performance.

Based on the nature of these interactions, MESs can use processes that involve: (i) extracellular electron transfer (primary MES) in which the electrode potential lies within the physiological range of the microorganisms and rely primarily on Faraday processes (e.g., microbial electrocatalysis through extracellular electron transfer) and, (ii) indirect interactions in which the microbial environment (metabolite, pH, oxygen pressure, etc.) is controlled by electrochemical processes [3]. For these processes to take place, the microbial system should be in close vicinity of the electrochemical system and the system needs to be integrated into a reactor that ideally would require low operation and maintenance cost. The electrode surface, microbial kinetics, reactor configurations and the electrogenic microorganisms used to construct the device all play a critical role in controlling these processes. In the past several years, there have been advancements in the development of new materials for bioelectrodes, engineered microbes, substrate types and interspecies electron processes to improve interfacial electron transfer and the microbial/electrode interface [4]. While these new developments have improved performance, several challenges still exist that hampered the implementation of these systems in real world applications. This article reviews the principles of MES, their development status and promise for implementation in the biosensing field.

## 2. Principles of Microbial Electrochemical Systems

Microbial electron transport chain (METC) represents the most important compartment in the living systems, since the oxidation of degradable organic substrates is the main energy source of live microbial cells [5]. Therefore, measuring the efficiency of microbial respiration and the activity of the electron transport chain are considered main indicators of cellular activity, as they are essential for the replication and proliferation of aerobic organisms [6]. Hence, earlier efforts were made to employ the measurement of dissolved oxygen consumption by living cells as a direct measure of the microbial survival [7]. Consequently, the electron transfer process from living-microorganisms towards electrodes in MES is exploited in microbial fuel cells [8,9] or diagnostic tools for rapid assessment of microbial activity [10,11,12]. In these regards, many MES approaches were designed and tested for biological purposes [13,14,15,16]. The electrical current value generated by the MESs is directly proportional to the number of viable microbial cells that are incorporated in MESs. On the other hand, non-viable or non-cultivable living cells do not have electrochemical contribution, and thus, they do not generate electrochemical signals. Thus, the resulting bioelectrochemical responses reflect the extent of anodic respiration, intracellular redox reactions (e.g., intracellular enzyme activities) and/or other biological interactions [17,18]. Since the bioelectrochemical responses can be linked to microbial processes, the design of high performance MESs has gained increasing attention due to their many promising applications in the environment, energy and biomedical fields.

### 2.1. Extracellular Electron Transfer

**Figure 1:**
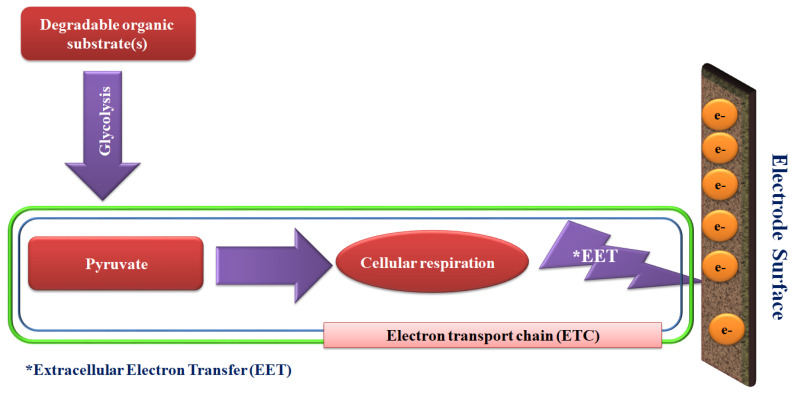
Basic concept of microbe-electrode interactions in common microbial electrochemical systems. Adhered living microbes transfer the biological produced electrons to the electrode surface to generate bioelectrochemical signals.

The operation of primary MES involves extracellular electron transfer (EET), directly from the cell to the acceptor, or indirectly mediated by electron shuttles. An example of bioelectrochemical signals generated in the MES systems is shown in Figure 1, illustrating conversion of degradable organic substrates to pyruvate via the glycolysis process, which is the central precursor of generating bioenergy.

**Figure 2:**
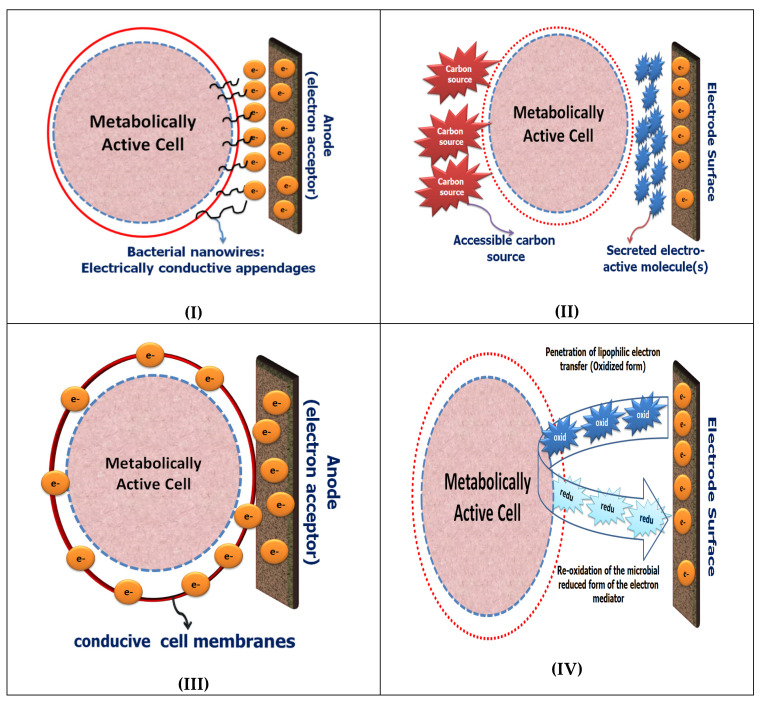
Illustration of the Extracellular electron transfer in the microbial electrochemical systems. The direct microbial electro transfer (DET) is enabled through the growing of nanowires (**I**), or by the formation of a conductive layer at the microbial cell wall (**III**). The mediated electron transfer (MET) was conducted either via the naturally secreted mediators (**II**) or by the chemically utilized electron redox-shuttles (**IV**).

In the absence of oxygen, as the final electron acceptor, anodic respiration is the main regulating factor for the electron transfer from the living cells to the electrode surface. Through the classical aerobic respiration pathway, two electrons liberated by the enzymatic oxidation of NADH or NADPH via the first enzymatic complex NADH dehydrogenase and transferred to coenzyme Q (CoQ), to ubisemiquinone, and then to ubiquinol (the natural electron shuttles). Subsequently, the electrons from ubiquinol are transferred through the electron transport chain (ETC) to complex III (*bc1* complex), cytochrome *c*, complex IV (cytochrome *c* oxidase), and finally to oxygen (as the final electron acceptor) to produce H_2_O. The energy that is released, due to the electrons flow down the electron transport chain, is used to pump protons out through complexes I, III, and IV. This creates a proton electrochemical gradient [19]. Keeping in mind that the electron transport chain is physically separated from the outside environment by cytoplasmic membranes with additional layers, such as cell walls, peptidoglycans, or outer membranes the physical transfer of the biologically liberated/created electrons to the surface of the electrode also plays an important critical role [20,21]. Mechanisms for transferring electrons from the microbial intracellular compartments to the surface of the electrode have been studied predominantly in bacterial systems; thus the use of bacteria is more common in the construction of microbial fuel cell technology. As shown in Figure 2, two different mechanisms can be used for connecting the living microbial cells with the electrode surface through direct electron transfer (DET) or mediated electron transfer (MET). The full description of each mechanism is given in the next section.

#### 2.1.1. Direct Electron Transfer via Electroactive Microbes

Through anodic respiration, certain microbial species, known as Exoelectrogens or Electroactive microorganisms, have been identified as electroactive organisms [22]. The *Exoelectrogens* usually refers to microorganisms that have the capability to transfer electrons extracellularly to conductive materials directly without mediators [23,24]. These electroactive microbes are also called anode respiring bacteria, electrochemically active bacteria, and electricigens [25]. Direct electron transfer (DET), which requires physical contact between the surface of electrode and the redox centers of microbes [26], can be achieved directly (mediated-less) via the electroactive species, e.g., cell-wall containing cytochromes, of the adhered organism [27,28,29,30,31]. Alternatively, a conductive layer in outer-membranes can form to conduct electrons [32]. For example, bacterial nanowires (*Pili*) were created by *Shewanella oneidensis* MR-1 in its outer membrane that facilitate extracellular electron transport [33,34,35,36]. Secretion of electroactive metabolite(s) in the extracellular matrix was also identified as an alternative mechanism to provide self-mediation of electron transport [26,37,38,39]. For example, microbial natural-electron mediators (water-soluble compounds with low molecular weight), which act as quorum sensing molecules, produced by *Shewanella putrefaciens* into the extracellular matrix, have shown the ability to mediate the electron transfer [40,41,42,43]. Phenazines, as the quorum sensing (QS) molecules, produced by Pseudomonas aeruginosa modulated the current production as a result of the anodic-respiration [44]. The DET is not limited to bacteria. Some eukaryotic microorganisms were also identified as potential candidates for that purpose, as illustrated by a mediated-less bioelectrochemical approach for studying the intracellular level of *Candida albicans* [45]. The electron transfer capacity was strongly controlled by the mitochondrial respiratory chain efficiency, since inhibition of the respiratory chain, either chemically using specific enzyme inhibitors, or genetically by knocking out specific genes from respiratory chain complexes, led to a decrease in the generated bioelectrochemical response [45].

#### 2.1.2. Mediated Electron Transfer

Unlike the *exoelectrogens*, many microorganisms are electrochemically inactive which means they are unable to transfer their electrons to the electrode surfaces without the use of soluble chemical redox mediators [46,47]. Hence, artificial redox mediators (exogenous electron shuttles) are required to enable the microbe-electrode interaction(s) through a process which is known as mediated electron transfer (MET) [43,48]. The MET is commonly used in the developments of MESs, and its redox reaction mechanisms are generally well understood; therefore, MES has been used for the rapid detection of cell viability and cytotoxicity [49,50,51,52]. For example, 2,6-dichlorophenolindophenol (DCIP) has been exploited as a traveling electron mediator (a membrane permeable molecule) for monitoring the cell viability of *Candida* and *Saccharomyces* species [20]. The specific action of DCIP, i.e., accepting the liberated electrons from the complex I of the respiratory chain system (mitochondrial NADH-dehydrogenases), which is not provided in the respiratory chain system of the *S. cerevisiae*, was a potential indicator for the selective assessing of complex I activity in the *C. albicans*. In another study, a double mediator approach using a mixture of *2*,*3*,*5*,*6*-Tetramethyl-p-phenylenediamine, or menadione in a combination with the hexacyanoferrate(III) (ferricyanide or FCN), was used to probe the intracellular redox activity and tracking the metabolic pathway activity of Chinese hamster ovary cells or *S. cerevisiae* [53,54]. As the double mediator systems were applied, fast electron transfer along with higher sensitivity were achieved when they were compared with the responses of the single mediator system. Accordingly, another double-mediated approach using DCIP/FCN has been developed and applied for probing the intracellular redox activity of *Staphylococcus aureus* and its pathway stimulation with different organic compounds such as glucose of acetate as a sole source of electrons. When only a single hydrophilic mediator such as the FCN was used, bio-electrochemical responses were not generated, indicating that the living bacterial cells are not communicating with the electrode surface. On the other hand, when the lipophilic mediator (DCIP) was used with the FCN, strong bio-electrochemical signals were generated, showing the possibility of wiring the living cells with the solid conductive surfaces. The double-mediator system was used to amplify the electric-current response from the intracellular NAD(P)H:quinone acceptor oxidoreductase (NQO) activity [34]. Therefore, the current magnification enhanced the assay performance [33]. A short summary of the direct and mediated ET between living microorganisms and the electrode surface is sketched in Figure 2.

## 3. Bioelectrochemistry of Biofilms

Basically, in the non-mediated microbial electrochemical systems, metabolically active microorganisms and electrodes are directly communicating together through the formation of electro-active biofilms [4,11,55,56]. In fact, the biofilms are a mixture of heterogeneous communities of microbial cells surrounded by a condensed layer of exopolysaccharides matrix (EPSs), and are strongly adhered to living tissues or solid surfaces [57,58]. In most biofilms, the living cells represent less than 10% of the total content in prevalence represented by the matrix (about 90%). In general, the biofilm matrices contain components such as polysaccharides, proteins and extracellular DNA, but their content is dependent on the bacterial species and the environmental conditions [59]. Moreover, it provides high mechanical stability, the EPS environment mediates the cell-cell adhesion to the solid surfaces and forms a cohesive three-dimensional network that interconnects biofilm cells.

The process of biofilm formation is of high importance in many industrial applications such as the biodegradation of chemical contaminants in wastewater, biocatalysis, and microbial fuel cells [18,60,61]. However, other biofilms may have serious implications on public health and environment [62]. For instance, microbial contamination on metal implants and prosthetic biomedical devices causing biofilm formation can be life-threatening, leading to chronic infections, device failure, and high mortality rates [63,64]. In the microbial electrochemical systems different biofilms could be characterized as electrochemically active or inactive [65]. The electrochemically active biofilms are defined as a community of microorganisms interacting with a conductive surface by either transferring to (anodic behavior) or removing electrons from the electrode (cathodic behavior) [66,67,68].

The nature of the biofilm assisted-bioelectrochemical signals was reported as physical connections through the bacterial appendages, microbial nanowires, cyt-c and ferric iron in some microorganisms such as *Thiobacillus denitrificans, Shewanella oneidensis* and *Geobacter sulfurreducens* [69,70]. O’Toole et al. reported the important role of flagella, and/or motility, in the initial cell-to-surface signal transduction [71]. They showed that defective flagella mutants (flgK) did not develop microcolonies on solid substrates over the course of the experiments. This observation supported the role of intact flagella to the microcolony formation. 

**Figure 3:**
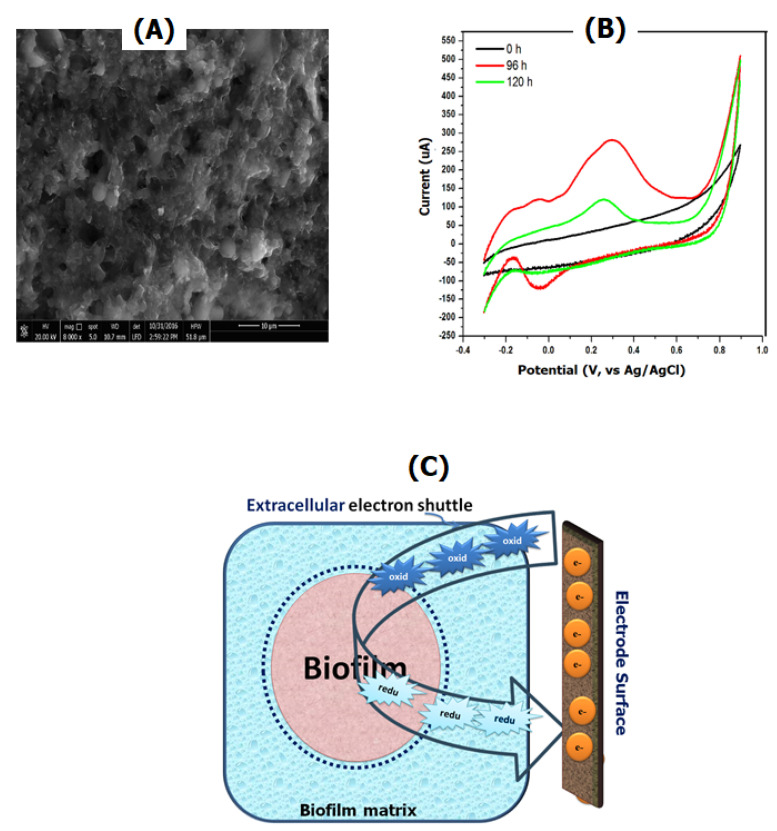
Characterization of biofilm formation at screen-printed electrodes using scanning electron microscopy and cyclic voltammetry. (**A**) Biofilm formation of bacterial mixed culture at screen printed electrodes modified with Multiwalled Carbon Nanotubes. (**B**) Direct bioelectrochemical signals of the formed biofilm at the SPE. (**C**) Mediated electron transfer used to monitor the bio-electrochemical responses of proliferated biofilms at electrode surfaces.

MESs were shown to be the most effective way for studying and understanding the role of the conductive solid surfaces and the environment in biofilm formation [4,72,73]. Bioelectrochemical analysis of the biofilm formation at different electrode modifiers was conducted to understand the influence of the electrode materials on the biofilm progression [11,74]. In addition to morphological characterization using microscopic techniques, cyclic voltammetry (CV) is another technique that can be used for monitoring the online formation of biofilms and their electrochemical activity [75,76]. Several redox peaks were observed over different incubation times with the bacterial culture, indicating a direct electron transfer from the outer-redox layer of the bacteria to the electrode surface. CV can be used in conjunction with physical characterization tools, e.g., scanning electron microscopy, to confirm the mature biofilm formation at the electrode surface as shown in Figure 3A,B.

On the other hand, electrochemically inactive biofilms could be integrated with electrode surfaces via extracellular electron receptors/transmitters, or electron mediators. Electron transmitters in the oxidized form can penetrate the cell-wall, as well as the cell membranes to capture the intracellular electrons. Afterwards, the reduced electron mediators are released to convey the accepted-electrons via redox reactions taking place at the electrode surface (as shown in Figure 3C). The use of soluble redox mediators in the MES bioreactor is an effective way to improve the extracellular electron transfer [77].

## 4. Microbial Electrochemical Devices

### 4.1. Half-Cell Based MESs

Construction and evaluation of the performance of MESs could be achieved by using CV, electrochemical impedance spectroscopy (EIS), or by amperometric measurements [78,79]. Those electrochemical techniques can be used to obtain mechanistic or kinetic information of extracellular electron transfer from living cells to the electrode surface and further the basic understanding of the microbe-electrode interaction [80]. Particular interest is always given to CV, since this method provides information about the formation of biofilms and their electrochemical and electro-catalytic activities, extracted from the real-time recording of voltammograms [81]. CV was used to characterize the anaerobic growth of *E. coli* and its secreted mediators and evaluate their role in the functioning of the cell, after the formation of a biofilm on the surface of the platinized titanium mesh electrodes [82]. Moreover, the biofilm formation and microbial adherence to the electrode surface could be effectively measured by using the impedimetric signal [79]. Additionally, monitoring the microbial cell number or microbial responses to different stresses could be determined from amperometric measurements [83]. Internal resistance variation during bacterial growth at electrode surface was assessed using EIS, whereas the equivalent circuit-based analysis revealed that the initial internal resistance of the cell has been internally reduced by around 50 % over an eight hour period of microbes-electrode incubation [84,85]. EIS is among one of the most powerful material characterization techniques for analyzing microbial-electrochemical reactions, monitoring biofilm progression, and evaluating microbial immobilization on different electrode materials. It can also be used for assessing the electrode properties and for studying the mass transfer resistances and evaluating the diffusion limitations of the reactants [86].

### 4.2. Microbial Fuel Cells

**Figure 4:**
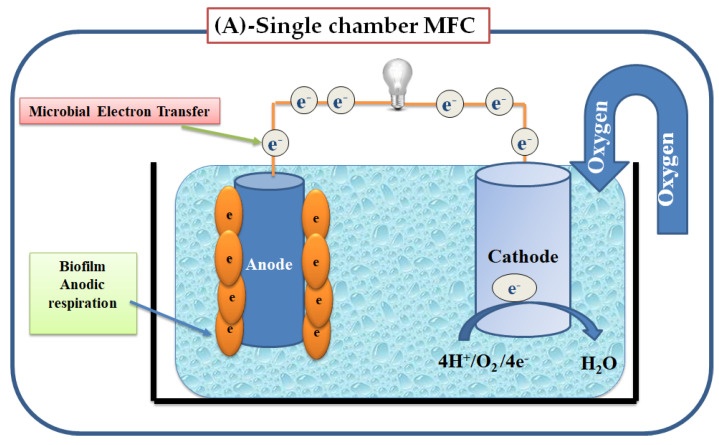

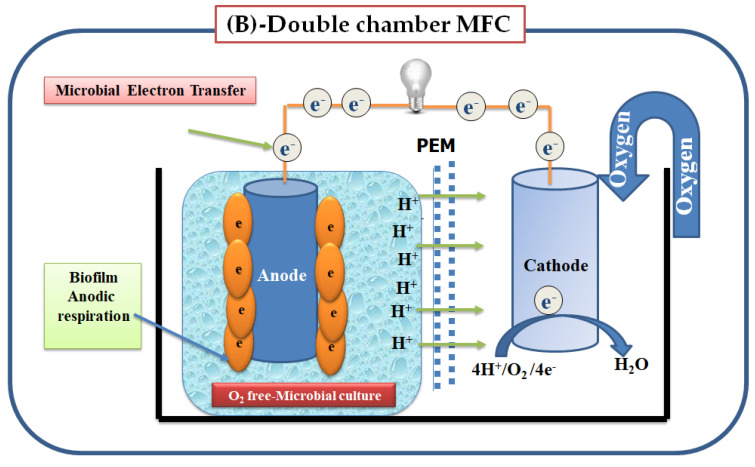
(**A**)**:** Design of single chamber MFC. (**B**)**:** Design of double chamber MFC separated by a PEM membrane. PEM stands for proton exchange membrane.

Microbial fuel cells (MFCs) are common microbial electrochemical systems that exploit the activity of living-microorganisms to convert chemical energy through the oxidation of organic substrates to electricity [87]. Typical MFCs consists of an anode and a cathode both of which are incubated with a liquid culture of living microbes (electrogenic or electroactive organisms). For the anodic reaction, living cells consume the degradable organic substrate (electron donors) that are transferred from the bulk solution and through the cellular metabolism to the anode surface [6]. Two different designs (i.e., single or double-chambers) of MFCs have been used, illustrated in Figure 4. In the double-chambers, an anaerobic anode chamber and an aerobic cathode chamber, are generally separated by a Proton Exchange Membrane (PEM) such as Nafion or sulfonated poly-(ether-ether-ketone) (SPEEK) as a kind of the non-fluorinated membranes [88]. Under hypoxic conditions, the anodic respiration followed by biofilm formation at the MFC electrode [89]. A mixed culture or single microbial strain could be used to build a MFC with high performance [90,91]. Regulator factors, biofilm structure adaptation, and/or the type of the microbial community have a great impact on the electrochemical characteristics. The number and dimensions of the biofilm pores affect the transfer of metabolites to the electrode surface while the transfer of protons, substrate and metabolites between the electrode surface and the bulk solution has an effect on the current generation [92]. The current generated by MFCs arises from the transfer of electrons received by the anode when live-bacteria oxidize organic materials, which are then transferred to the cathode via the external circuit, as shown in Figure 4.

Typically, MFCs are classified into two types depending on how extracellular electrons are delivered from the attached microorganisms to the anode; (i) Mediator-based MFCs, in which electro-active secreted metabolites, or artificial redox compounds are used to shuttle the electrons [93]; (ii) mediated-less fuel cells which does not require the addition of electroactive metabolites to transfer the electrons, but it relies mainly on the presence of electro-active organisms such as *Shewanella* [94,95], *Rhodoferax* [96] and *Geobacteraceae* [97]. These organisms transfer electrons directly to the anode via molecular nanowires and electrochemically active redox enzymes in their outer membrane. Wastewater treatments coupled with clean energy production are the most important practical applications of the MFCs [98]. The power generated by MFCs depends on various factors such as the electron transfer rate from the bacteria to anode, the diffusion of substrate into the biofilm, the ohmic resistance of the electrolyte and the electrochemical kinetics. The maximum power generated by the MFC also depends on the total internal resistance of the system [99,100]. The reviews of the basic MFCs technology, challenges and applications can be found in several articles [101,102].

## 5. Biosensing Applications of MESs for Microbial Detection

### 5.1. State of Art Pathogenic Microorganism’s Detection

Microbial infections have significantly increased over the past few decades and are now considered as one of the most critical global challenges. In particular, pathogenic microorganisms are responsible for bacterial infection which significantly affects the health of human, animals and plants. The detection of pathogens, including contaminants and other important harmful bio-molecules, such as toxins, plays a crucial role in the prevention of microbial infections. Rapid and sensitive detection with selective identification of microorganisms, including pathogens, are exceedingly important in clinical microbiology, microbial forensics, food and environmental analysis. However, the limited availability of efficient diagnostic tools is actually an impediment for rapid detection and treatment [103].

Classical microbiological methods widely used for the detection of pathogens, including culturing, plating techniques, microscopy, and serology, are insufficient for today’s needs. In particular, microscopy, which is a simple and relatively easy technique to be handled, lacks sensitivity. On the other hand, complicated steps, such as pre-enrichment, selective plating, biochemical screening and serological confirmation, are needed for the culture and plating methods [104]. Since such methods depend on the ability of microbes to grow and form colonies, its use is often time-consuming, tedious and unsuccessful [105]. Another widely used test for the microbial detection is the Enzyme-linked Immunosorbent assay (ELISA) that relies on the specific binding of antibodies to their specific antigens. ELISA allows the detection of very small quantities of antigens, such as molecules (hormones, toxins, etc.) and macromolecules (peptides, proteins, etc.) through a series of binding events [106]. Although largely used, this method requires intensive labor and, besides being expensive, is affected by false positives due to non-specific antigen-antibody reactions. To overcome these limitations, other molecular techniques, including DNA sequencing, Polymerase Chain Reaction (PCR) or microarray analysis, have been implemented, allowing a more sensitive and specific detection of microbes [107,108]. In spite of advantages in sensitivity, these molecular techniques still have limitations, related to the use of complicated protocols (requiring skilled workers to carry out the tests and their data interpretation) and of expensive materials (i.e., fluorescent probes for labeling). Moreover, all molecular methods cannot distinguish between viable and nonviable organisms which may lead to overestimation of infection [109].

### 5.2. Electrochemical Biosensing Devices

Biosensors have been proposed as alternatives to the current analytical methods, offering higher sensitivities, lower cost and portability for on-site analysis and multiplexing. Biosensors have attracted significant interest due to their potential importance for clinical, diagnostic, environmental, and bio-security applications [110,111]. Biosensors integrate biologically active species (biological recognition elements) with a physical transducer which converts the biological responses to measurable signals. These signals can be detected optically, acoustically, mechanically, calorimetrically, or electrochemically [112]. Electrochemical biosensors are the most common biosensing techniques due to their high specificity and sensitivity, and their ease of use. Also, their use allows the on-line detection of a broad spectrum of analytes in complex matrices (e.g., blood, serum, urine or food) [113,114]. Moreover, the advanced miniaturization of modern microelectronics allows building micro/and nano-electrodes, which are suited for detection of very small volumes of samples (microliters to nano-liters) [115]. The low cost and the possibility for large-scale production of electrochemical sensors, with pocket and portable devices, are other reasons, which make the electrochemical approaches more appealing for high-throughput analysis [85]. Several factors should be taken in consideration when developing biosensors to achieve high performances comparable or better than the other affordable methods:

1. Selection of electrode materials and surface modification before using as support for the immobilization of the bio-recognition element (i.e., enzymes, antibodies, peptides, aptamers, phages or whole cells) [116].
2. The selection of the electrochemical method (e.g., potentiometric, voltammetric, amperometric, or impedimetric method).

Electrochemical immunosensors are among the most common platforms for microbial detection and identification [117,118]. The signal is usually obtained through the detection changes of an immobilized recognition element (i.e., antigen or antibody) on the electrode surface. Two possible mechanisms can be used to develop an electrochemical immunosensor, based on a competitive or a sandwich organization. When dealing with competitive immune-sensors, two approaches could be considered: (1) Immobilizing antibodies, which react with free antigens from the sample in competition with labeled antigens, returning the amount of unreacted antibodies and indirectly the amount of sample antigens; and (2) immobilizing antigens, and using labeled antibodies to measure the amount of unreacted antigens. This last approach is useful to prevent problems related to antibody immobilization (loss of affinity, orientation of the immobilized protein) [118]. In a sandwich assay, using immobilized antibodies, labeled secondary antibodies (directed toward a second binding site of the antigen) are added. At this point, antigen is “sandwiched” between two antibodies. In spite of the high sensitivity and selectivity, antibodies have limited stability and tend to denature in conditions outside the physiological range. Among different alternative approaches, biomimetic artificial receptors having superior stability have been explored [119,120]. Molecularly imprinted polymers (MIPs), defined as artificial recognition elements, are of growing interest for applications in several sectors of life science for detecting molecules of specific interest MIPs can be developed to have high bio-recognition capability, mechanical and chemical stability. Also, they are relatively easy to prepare and have low cost, which make them superior over natural recognition reagents [121].

### 5.3. Biosensing Applications of MESs

**Figure 5:**
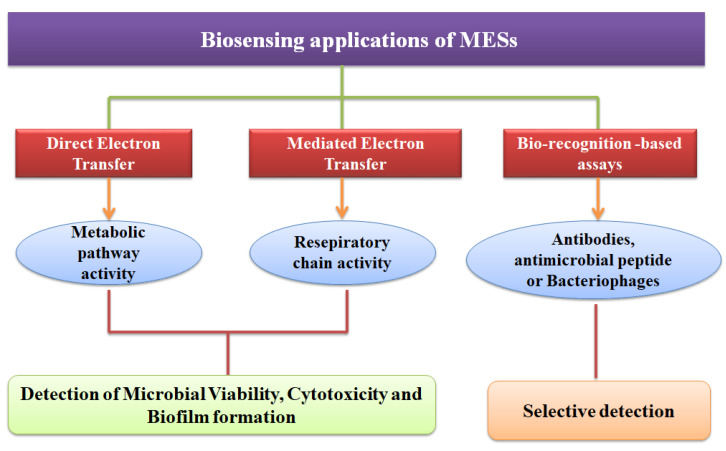
Application of microbial electrochemical systems based on monitoring of microbial cell viability, intracellular activities, biofilm formation.

Instead of applying biological recognition elements, such as antibodies, the online-measuring of cell viability with the ability to distinguish living from the dead cells can be achieved through the use of MESs [1,12]. MESs could be used effectively to distinguish between live and dead microbial cells, monitor the intracellular redox functions of living cells, and characterize electrochemically active biofilms. Because the construction of MESs involves the effective integration of microbiology with electrochemistry and material sciences, major improvements in the operation of MESs can be achieved by optimizing the electrode materials to improve the electron transfer at the microorganism-electrode interface [122]. A summary of the most common biosensing applications of the MESs is provided in Figure 5, and details were expanded in Table 1.

### 5.4. Impact of the Electrode Materials on the Performance of MESs

Conventional MESs were built using carbon materials, such as graphite granules, graphite felt, carbon paper, and carbon cloth [128,129]. Inherent drawbacks of these materials due to the limited surface area have been addressed by exploring the benefit of nanostructured materials, which can provide enhancements of the performance of MESs by virtue of their specific high surface areas, conductivity and catalytic activity. In particular, carbon-based materials, including carbon fibers, activated carbon, graphene, carbon nanotubes (CNTs), and fullerene exhibit high electrochemically active surface area [130,131] that can be used as electrode materials for enhancing the detection limits, and advance the development of new MESs-based detection technologies [132,133,134].

In MESs, the microbial kinetics such as oxidation of the extracellular secretions or the metabolic activities of microorganism are affected by the reactions taking place at the electrode surface [135]. A wide range of materials have been used as electrode materials, e.g., carbon paper, cloth, graphite foil, rods, metal or metal nanoparticles to modify working electrodes. The material used can significantly alter the electrocatalytic functions, accelerate the biofilm formation, or catalyze the secreted biomolecules, which can facilitate the electron transfer process [136]. By integrating nanomaterials, more effective microbial sensors were developed, demonstrating promise of this technology for real applications [137].

Nanostructured materials, such as metal and metal oxide nanoparticles (NPs), or nano-hybrids have been employed in the construction of MESs [138]. Carbon nanostructures have shown promising performance as electrode material, due to their high electrical conductivity, biocompatibility, and electrocatalytic properties [139]. Owing to its large surface area, rich electronic states, and good mechanical properties, graphene-based materials have played an important role in the development of MESs in recent years. Graphene offers a large surface area for bacterial colonization, facilitates direct electron transfer and improves the electron transfer efficiency [140]. Graphene has been used in the manufacturing of MESs due to its physicochemical properties, biocompatibility, mechanical strength and flexibility [141]. A significant enhancement for the bacteria-electrode interactions was observed at graphene-modified electrodes and thus the measurement time has been reduced and the assay sensitivity has been improved [142]. Graphene stimulated the microbial production of phenazine, a well-known electron shuttling quorum sensing molecule for the extracellular electron transfer process in *Pseudomonas aeruginosa* [143]. The use of a macroporous monolithic MFC anode based on polyaniline hybridized three-dimensional (3D) graphene was effective at addressing problems related to the low bacterial loading capacity and low extracellular electron transfer efficiency between the bacteria and the electrodes [144,145]. Flexible 3D reduced graphene oxide–nickel (rGO–Ni) foam was used to increase the power generation of microbial fuel cells [146]. Other carbon materials such as CNTs and multi-walled carbon nanotubes (MWCNTs), especially when functionalized with chemical groups like amines or carboxylates were shown to lower the over-voltage and facilitate detection and fast electron transfer rates, leading to higher sensitivity of microbial detection [147]. For example, a MWCNTs/CPE sensor exhibited better electro-catalytic performance and direct electron transfer capability for detection of metabolically active bacterial cells [148]. In addition, it has been reported that CNTs provided a suitable platform for a cell adhesion, cell attachment and growth. Tsai et al. coated CNTs over a carbon cloth to form a highly conductive MFC anode with a large surface area and found that the maximum power density improved by 250 percent [149]. A dispersion of nanocrystalline platinum anchored CNTs in water was used in a two-chambers MFC to accelerate the mediated electron transfer [150]. In a report by Sharma et al. the electron transfer efficiency of the *E. coli*-based bioelectrochemical system was enhanced by electrode modification with the CNTs [151].

In addition to carbon-based nanomaterials, metal NPs (MNPs) have also shown promise for the construction of MESs due to their superior conductivity, large surface area and high catalytic activity. Therefore, the decoration of electrodes with metal nanostructures is an effective way to enhance direct electron transfer and the catalytic activity of the working electrodes [123]. In 2014, Lieber group showed that biogenic NPs can serve as “bridges” to facilitate efficient extracellular electron transfer from *Shewanella* cells to electrode surfaces and also between interconnected cell networks [152]. Au–Ag core–shell NPs were used by Ding and colleagues to form aggregates for simultaneous bacterial imaging and synergistic antibacterial activity [125]. In addition, the power generation of MFC was improved with AuNPs modified carbon paper [153]. On the other hand, metal oxide nanostructures were utilized in mi sensors especially in the form of nanocomposites with MNPs or other nanomaterials [154]. For example, ZnO formed nanocomposite with AuNPs, graphene, MWCNTs, and these hybrids were used for the construction of high performance MESs [100,143].

Owing to their unique characteristics such as high surface-to-volume ratio, electrocatalytic activity, electrical and mechanical properties, nanomaterials have intensively studied for use in MESs particularly for accelerating the electron transfer [11,155,156] and promote microbial-electrode adherence and biofilm formation [138,157].

## 6. Conclusions and Future Perspectives

In this article, the use of MESs was reviewed as a potential non-destructive monitoring tool for assessing microbial cell viability, biofilm formation and intracellular redox functions. A comprehensive discussion of the developments of the first and second generation of MESs was provided along with an overview highlighting recent developments in the field such as the use of nanostructured materials to improve electrodes performance and the integration of MESs with spectroscopy techniques. Due to the high conductivity, large surface area, and low interfacial charge resistance provided by most of the most nanostructured electrodes, significant improvements in the microbe-electrode interactions and in the extracellular electron transfer process can be achieved using nanomaterials as electrode materials. To achieve practical implementation, several future developments are needed. First, there is a need to improve the overall performance (e.g., power generation, stability and component integration) and reduce the cost of MESs for these to provide a competitive solution to existing technologies. Second, MESs need to be scaled up and efforts need to be dedicated to integrate the existing configurations to address current needs and reach out detection limits that are of practical relevance. Third, performance of these devices in practical scenarios and side-by-side comparison with accepted methods should be evaluated to demonstrate feasibility for implementation. From a fundamental perspective, integration of MESs with other techniques, such as scanning tunneling microscopy (STM) and/or atomic force microscopy (AFM) may provide greater insight about the structural behavior of the microbes at electrode surfaces and other electroactive (intra or extracellular) species. This could help achieve better understanding of the molecular structures, electron transfer mechanisms, and the interactions between microbes and electrodes.

## Figures and Tables

**Table 1:** Comparison of classical methods and MES-based techniques for microbial detection.

**Classical Microbiological Methods**					
**Techniques**	**Test Based on**	**Detection**	**Advantages**	**Disadvantages**	**References**
Plating techniques	Culturing	Coloration Fluorescence Selective plating	direct detection	time-consuming serological confirmation fail to detect non-cultivable bacteria	[104]
Microscopy	Optical	Coloration Fluorescence	simple and relatively easy techniques	lack sensitivity	[123]
Serology	Antibodies/Antigens	Precipitation Coloration Fluorescence	detection of very small quantities	requires intensive labor expensive affected by false positives	
PCR/DNA sequencing	Genetic—Molecular	Sanger methodology Dye labeled nucleotides	high sensitivity	complicated protocols require skilled workers expensive materials overestimation of infection	[107,108]
Microarray	Molecular	Fluorescence	high sensitivity	complicated protocols require skilled workers expensive materials overestimation of infection	[124]
**MES Biosensors**					
**Organisms**	**Test Based on**	**Detection**	**Applications**	**Advantages**	
*Candida albicans*	DCIP ^1^	Electrochemically Photometrically	measure cell viability	distinguish between viable and nonviable organisms	[20]
*Staphylococcus aureus*	FCN ^2^ DCIP ^1^	Electrochemically	monitor cell proliferation estimate the cell number measure cell viability probe redox centers	rapid detection distinguish between viable and nonviable organisms	[125]
*Saccharomyces cerevisiae*	FCN ^2^	Electrochemically	locate cellular source of electrons	study cell pathways monitor the intracellular redox functions of living cells	[53]
*Saccharomyces cerevisiae*	Menadione ^3^ DCIP ^1^ TMPD ^4^	Electrochemically	biotoxicity assay	monitor the intracellular redox functions of living cells	[53,54]
*Pseudomonas aeruginosa*	- DET ^5^	Electrochemically	monitor microbial attachment monitor biofilm formation	monitor the intracellular redox functions of living cells characterize electrochemically active biofilms	[18,126]
Bacterial Growth	- DET ^5^	Electrochemically	Monitoring of Bacterial Growth monitor chemical oxygen demand	Online Monitoring of Bacterial Growth	[127]

^1^ 2,6-dichlorophenolindophenol; via oxidation reactions of DCIP; ^2^ ferricyanide; ^3^ 2-methyl-1,4-naphthalenedione; ^4^ N,N,N′,N′-tetramethyl-p-phenylenediamine. ^5^ DET is standing for the direct electrochemical testing.
